# Folic Acid-Terminated Poly(2-Diethyl Amino Ethyl Methacrylate) Brush-Gated Magnetic Mesoporous Nanoparticles as a Smart Drug Delivery System

**DOI:** 10.3390/polym13010059

**Published:** 2020-12-25

**Authors:** Abeer M. Beagan, Ahlam A. Alghamdi, Shatha S. Lahmadi, Majed A. Halwani, Mohammed S. Almeataq, Abdulaziz N. Alhazaa, Khalid M. Alotaibi, Abdullah M. Alswieleh

**Affiliations:** 1 Department of Chemistry, College of Science, King Saud University, Riyadh 11451, Saudi Arabia; 437204374@student.ksu.edu.sa (A.A.A.); 438204365@student.ksu.edu.sa (S.S.L.); khalid.m@ksu.edu.sa (K.M.A.); 2 King Abdullah International Medical Research Center, Nanomedicine Department, King Saud bin Abdulaziz University for Health Sciences, Riyadh 11451, Saudi Arabia; halawanima@NGHA.MED.SA; 3 King Abdulaziz City for Science and Technology, Riyadh 11451, Saudi Arabia; mmeataq@kacst.edu.sa; 4 Department of Physics and Astronomy, College of Science, King Saud University, Riyadh 11451, Saudi Arabia; aalhazaa@ksu.edu.sa

**Keywords:** magnetic mesoporous nanoparticles, pH-responsive polymer brushes, surface functionalization, controlled drug release, anti-cancer drug

## Abstract

Currently, chemotherapy is an important method for the treatment of various cancers. Nevertheless, it has many limitations, such as poor tumour selectivity and multi-drug resistance. It is necessary to improve this treatment method by incorporating a targeted drug delivery system aimed to reduce side effects and drug resistance. The present work aims to develop pH-sensitive nanocarriers containing magnetic mesoporous silica nanoparticles (MMSNs) coated with pH-responsive polymers for tumour-targeted drug delivery via the folate receptor. 2-Diethyl amino ethyl methacrylate (DEAEMA) was successfully grafted on MMSNs via surface initiated ARGET atom transfer radical polymerization (ATRP), with an average particle size of 180 nm. The end groups of poly (2-(diethylamino)ethyl methacrylate) (PDEAEMA) brushes were converted to amines, followed by a covalent bond with folic acid (FA) as a targeting agent. FA conjugated to the nanoparticle surface was confirmed by X-ray photoelectron spectroscopy (XPS). pH-Responsive behavior of PDEAEMA brushes was investigated by Dynamic Light Scattering (DLS). The nanoparticles average diameters ranged from ca. 350 nm in basic media to ca. 650 in acidic solution. Multifunctional pH-sensitive magnetic mesoporous nanoparticles were loaded with an anti-cancer drug (Doxorubicin) to investigate their capacity and long-circulation time. In a cumulative release pattern, doxorubicin (DOX) release from nano-systems was ca. 20% when the particle exposed to acidic media, compared to ca. 5% in basic media. The nano-systems have excellent biocompatibility and are minimally toxic when exposed to MCF-7, and -MCF-7 ADR cells.

## 1. Introduction

Cancer is one of the most common causes of death in the world [1,2,3]. Nowadays, surgery, radiation, and chemotherapy are widely used to treat most cancers. Surgery and radiotherapy are used for local and non-metastatic cancers. Yet, they are inefficient when cancer has spread throughout the body. Therefore, chemotherapy, where an anti-cancer drug used, is considered to be an effective treatment method. Anti-cancer drugs are toxic molecules that work to stop or slow the growth of cells, whether cancerous or healthy cells [4,5,6,7,8]. Thus, it is very important to develop a new drug delivery system to transport anti-cancer drugs, targeting tumour tissue [9,10,11]. Various drug delivery systems have been developed as a nanocarriers, such as a carbon nanotube [12], nanostructured polymers [13], and mesoporous silica nanoparticles [14,15,16].

Mesoporous silica nanoparticles (MSNs) have been utilized as a nanocarrier due to their low toxicity, chemically inert, easy chemical modification, and large loading capacity [17,18,19]. Magnetic mesoporous silica nanoparticles (MMSNs) have been paid attention due to their potential in medical applications, such as magnetic resonance imaging (MRI), magnetic bio-separations, and drug delivery [20,21,22]. Furthermore, a magnetic mesoporous nanocarrier could target the cancerous tissues inside the body when an external magnetic field is applied [23,24]. Siminzar et al., functionalized the surface of MMSNs with an aptamer (mucin-1) for the delivery of doxorubicin (DOX) to the breast cancer cells [25]. Cai et al., has reported the fabrication of superparamagnetic mesoporous silica nanoparticles coated with MDA-MB-231 cell membranes as a biomimetic drug delivery system [26].

It is necessary to modify the surface of MMSNs with organic molecules such as stimuli polymer brushes to obtain some required properties such as adhesion, charge density, and controlled drug release [27,28,29]. The end of the polymer chains, in brushes regime, are attached to the particles’ surface via physisorption or covalent attachment. Two techniques have been wildly used to grow polymer brushes: reversible addition-fragmentation chain transfer polymerization (RAFT) and atom transfer radical polymerization (ATRP). In stimuli-responsive polymer brushes, the chain conformation change when the surrounding environment has changed, such as light, temperature, and pH [30,31,32].

Many studies have been reported on the modification of mesoporous nanomaterials with pH-responsive polymers as gatekeepers [33,34,35]. Xu et al., loaded quercetin into silica nanoparticles coated with poly (2-(diethylamino)ethyl methacrylate) (PDEAEMA) brushes [36]. The results indicated that the nano-system could be used as potential anti-cancer drug carriers. Pourjavadi et al., synthesized MMSNs grafted with polymer brushes poly (N-isopropyl acrylamide-co-glycidyl methacrylate) (PNG) modified with hydrazine groups as DOX binding sites [34]. Our previous work reported the synthesis of mesoporous silica grafted with diblock brushes of 2-(tert-butylamino) ethyl meth acrylate-b-poly (ethylene glycol) methyl ether methacrylate as a doxycycline nanocarrier [37].

Most cancer cells are overexpressing some definite receptors on their surfaces. Thus, the surface modification of the nanocarriers with targeting ligands will have a remarkable effect on cancer treatment. The outer surface of mesoporous nanoparticles can be decorated with some targeting molecules such as hormones, antibodies, peptides, and vitamins. Folic acid (FA) could attach selectively to the folate receptor (FR), present on some cancer cells’ surface [38,39]. Niedermayer et al., have prepared mesoporous silica nanoparticles attached with pH-responsive poly (2-vinylpyridine) (PVP) and folic acid as a targeted molecule [40]. Folic acid-polyethylenimine (FA-PEI) coated mesoporous nanoparticles were synthesized as a smart drug carrier by Park et al. [41] Li et al., have developed mesoporous silica conjugated with a pH responsive polymer capped with folic acid [42]. It was observed that the fabricated nanoparticles exhibited high loading efficiency with zero pre-release of the drug within 20 h in normal surroundings. Wu et al., modified magnetic mesoporous silica nanoparticles with poly (ethylene glycol) (PEG) and FA to specifically target cancer cells [43]. They found that the presence of FA on nanoparticles’ surface increased the uptake into cancer cells that over-express FA receptors (FRs).

**Scheme 1:**
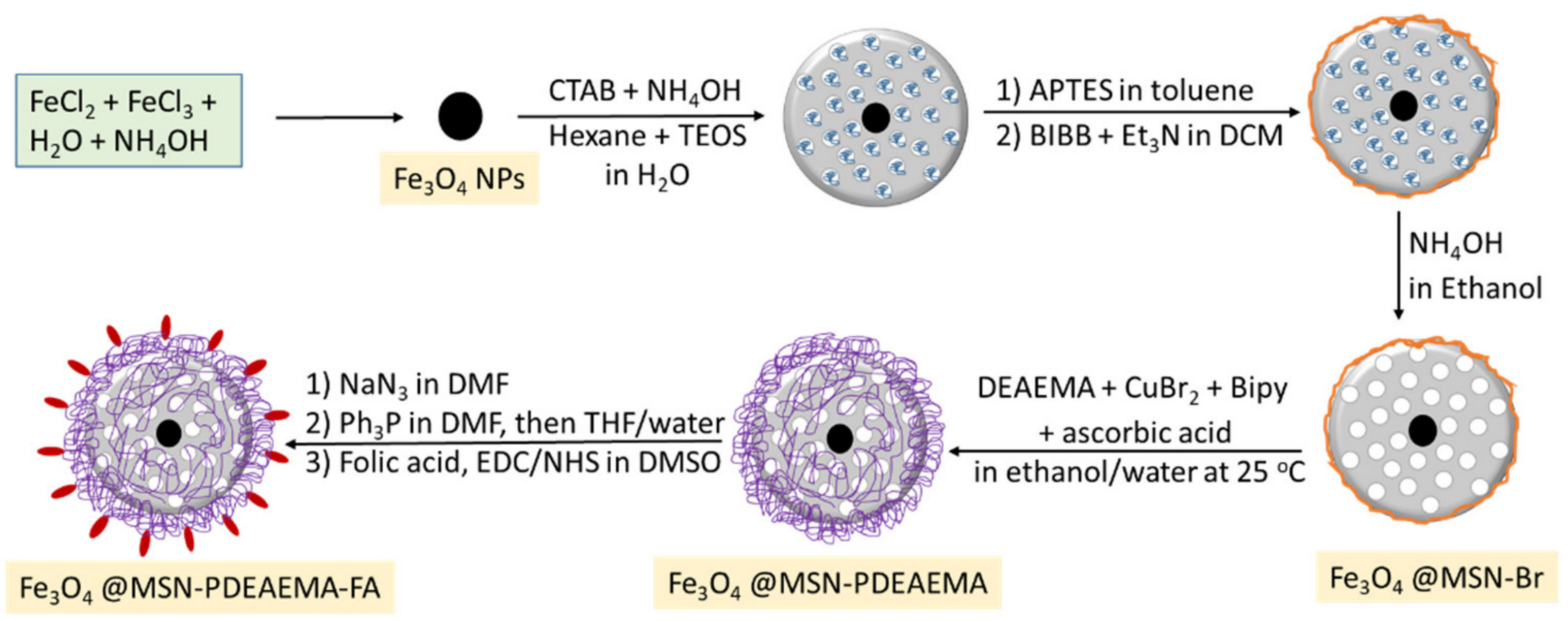
Synthesis procedure for poly(2-(diethylamino) ethylmethacrylate) brushes capped folic acid grafted on magnetic mesoporous silica nanoparticle (MMSNs) surfaces via SI-ARGET ATRP.

As far as we are aware, very little work has been reported on polymer brush surface modification with targeting molecules. This study aims to develop a new system consisting of targeting a ligand capped pH-responsive polymer grafted into the outer surface of the magnetic mesoporous nanoparticles. This can be achieved by derivatization of halogen groups on the surface of the polymer brushes with folic acid. In this study, magnetic mesoporous silica nanoparticles (MMSNs) were coated with pH-responsive poly (2-(diethylamino) ethyl methacrylate) (PDEAEMA) brushes via an ARGET ATRP technique. The end group of PDEAMA brushes was derivatized with folic acid (FA) for tumour-targeted drug delivery via the folate receptor, Scheme 1. The nanocarriers were loaded with Doxorubicin (DOX) and the release behavior was investigated. The nano-systems were subjected to MCF-7 and MCF-7 ADR cells for a cytotoxicity test. The characteristics of the samples were studied using various techniques such as scanning electron microscopy (SEM), transmission electron microscopy (TEM) and X-ray photoelectron spectroscopy (XPS).

## 2. Materials and Methods

### 2.1. Materials

Deionized water was obtained from Elga Pure Nanopore System. Ammonium hydroxide (28 wt %, CAS 1336-21-6), 3-aminopropyltriethoxysilane (APTES, >98%, CAS 919-30-2), N-cetyltrimethylammonium bromide (CTAB, 98%, CAS 57-09-0), methanol (99.8% HPLC grade, CAS 67-56-1), tetraethylorthosilicate (TEOS, 98%, CAS 78-10-4), ethanol (99.8%, HPLC grade, CAS 64-17-5), pyridine (analytical grade, CAS 110-86-1), dichloromethane (DCM, HPLC grade, CAS 75-09-2), 2,2′-bipyridyl (bipy, 99%,CAS 366-18-7), isopropanol (99.8%, CAS 67-63-0), 2-bromo-2-methylpropionyl bromide (BIBB, 98%, CAS 20769-85-1), (2-(diethylamino) ethylmethacrylate) (PDEAEMA, 99%, CAS 2867-47-2), triphenylphosphine (pph3, 99%, CAS 603-35-0), dimethylformamide (99.9%, HPLC grade, CAS 68-12-2), n-hexane (HPLC grade, CAS 68-12-2), sodium Hydroxide (CAS 1310-73-2), and folic acid (FA, CAS 59-30-3) were purchased from Sigma-Aldrich. Cupric bromide (CuBr_2_, 98%, CAS 7789-45-9) was purchased from BDH chemicals. Sodium azide (NaN3, 99%, CAS 26628-22-8), triethylamine (TEA, 99%, CAS 121-44-8), ferric chloride (99%, CAS 7705-08-0), and ferrous chloride (99%, CAS 7758-94-3) were purchased from Loba Chemie. Tetrahydrofuran (THF, CAS 109-99-9) were purchased from Nexgen chemicals. Ammonium nitrate (NH_4_NO_3_, 99%, CAS 6484-52-2) was obtained from Winlab chemicals reagents fine chemicals. N-Hydroxysuccinimide (NHS, CAS 6066-82-6), doxorubicin hydrochloride (DOX.HCl, CAS 25316-40-9), dimethylsulfoxide (DMSO, CAS 67-68-5), and N, N’-Di cyclohexylcarbodiimide (DCC, CAS 538-75-0) were obtained from Tokyo Chemical Industry. Sodium chloride (CAS 7440-23-5), potassium chloride (CAS 7440-09-7), disodium hydrogen phosphate (CAS 7558-79-4), potassium dihydrogen phosphate (CAS 7778-77-0), and hydrochloric acid (CAS 7647-01-0) were obtained at Alfa Aesar. All the chemicals were used as received.

### 2.2. Methods of Preparation

#### 2.2.1. Synthesis of Iron Oxide Nanoparticles (Fe_3_O_4_)

Iron oxide nanoparticles (Fe_3_O_4_) were synthesized according to previous published studies [44,45]. Ferric chloride (FeCl_3_, 4.80 g) and ferrous chloride (FeCl_2,_ 3.12 g) were dissolved in 30 mL of deionized water under nitrogen atmosphere with stirring at 90 °C. Ammonium hydroxide (20 mL) was added to the mixture and aged for 150 min. The product was filtered and washed with water and ethanol several times.

#### 2.2.2. Synthesis of Iron Oxide Coated with Silica (Fe_3_O_4_@MSNs)

Iron oxide coated with mesoporouse silica was prepared by suspending 0.5 g of Fe_3_O_4_ in 160 mL of deionized water, which was followed by adding 1.0 g of CTAB at 35 °C. Concentrated ammonia water (7 mL, 28 wt %) was added. Then a mixture solution of n- hexane (20 mL) and TEOS (5 mL) were added into the solution drop wisely within 30 min under continuous stirring. A homogeneous milky colloidal solution was gradually formed. After stirring for 12 h, the product was collected by filtration and washed several times with deionized water and ethanol. The materials were dried in an oven for 2 h at 100 °C.

#### 2.2.3. Synthesis of Amino Functionalized Magnetic Mesoporous Silica (Fe_3_O_4_@MSN-NH_2_)

Amino modification of the silica surface was performed by suspending the obtained (Fe_3_O_4_@MSNS) nanoparticles (1.5 g) in a solution of APTES (0.5 mL) and methanol (50 mL). The resulting mixture was heated at 70 °C for 12 h. The nanoparticles were collected by filtration and then washed several times with ethanol.

#### 2.2.4. Synthesis of the ATRP Initiator Functionalized Magnetic Mesoporous Silica (Fe_3_O_4_@MSN-Br)

Fe_3_O_4_@MSN-NH_2_ (1.0 g) was suspended in a mixture of DCM (25 mL) and triethylamine (1.5 mL). 2- Bromo-2-methylprpionyl bromide (1.2 mL) in 5 mL of DCM was added drop wise into the mixture and stirred at room temperature for 48 h. The solid was then separated by filtration and washed several times with DCM and ethanol.

#### 2.2.5. Formation of Channels (Fe_3_O_4_@MSN-Br)

The surfactant was removed by ionic exchange using a solution of ammonium nitrate (10 mg/mL) in ethanol (95%) at 70 °C overnight under stirring. The nanoparticles were collected and washed with ethanol.

#### 2.2.6. Synthesis of Poly(2-Dimethyl Amino Ethyl Methacrylate) (PDMAEMA) Brushes Coated Magnetic Mesoporous Silica (Fe_3_O_4_@MSN-PDMAEMA)

In small vial, Fe_3_O_4_@MSN-Br (0.4 g) was suspended in a degassed mixture of ethanol (4 mL) and deionized water (1 mL). A mixture of 2-dimethyl amino ethyl methacrylate (DMAEMA) (2 mL), CuBr_2_ (0.0009 g), and of 2,2 bipy (0.0067 g) dissolved in ethanol (8 mL) and water (2 mL) was added to the suspension and degassed for 15 min. Ascorbic acid (0.0076 g) was added to the polymerization mixture and stirred for 3 h. The solid was washed with 0.01 M of HCl solution and ethanol several times.

#### 2.2.7. Synthesis of (Fe_3_O_4_@MSN-PDMAEMA-NH_2_)

Fe_3_O_4_@MSN-PDMAEMA (200 mg) was suspended with 2 mL of degassed dimethylformamide (DMF) and kept under N_2_. Sodium azide (NaN_3_) was dissolved in degassed DMF to make a solution of 2 M. Sodium azide (NaN_3_) solution (5 mL) was added to Fe_3_O_4_@MSN-PDMAEMA under N_2_ atmosphere and 60 °C for 18 h.

Fe_3_O_4_@MSN-PDMAEMA-N_3_ (200 mg) was suspended with 2 mL of degassed dimethylformamide (DMF) and kept under N_2_. Triphenylphosphine (PPh_3_) was dissolved in degassed DMF to make a solution of 2 M. Triphenylphosphine (PPh_3_) solution (5 mL) was added to Fe_3_O_4_@MSN-PDMAEMA-N_3_ under an N_2_ atmosphere and 60 °C for 18 h.

The product was suspended in a mixture of water and tetrahydrofuran under nitrogen atmosphere for 18 h at 40 °C. The final product was washed with water and ethanol and dried at 60 °C for 2 h [46,47].

#### 2.2.8. Nanoparticles Modification with Folic Acid (Fe_3_O_4_@MSN-PDMAEMA-FA)

Folic acid (2.5 g) was dissolved in a mixture of DMSO (50 mL) and triethylamine (1.3 mL). DCC (2.3 g) and NHS (1.3 g) were added to the mixture and stirred over night at room temperature in the dark. The by-product is removed by filtration using glass fiber paper [48]. Fe_3_O_4_@MSN-PDMAEMA-NH_2_ (0.5 g) was added to 25 mL of the filtrated solution and stirred overnight at room temperature. The final product was filtered and washed with DMSO and ethanol.

### 2.3. Measurement and Characterization

Scanning Electron Microscopy (SEM) images were taken by JEOL JSM-7610F at 15 KV. Transmission Electron Microscopy (TEM) images were taken by A JEOL JEM-1400 at 100 KV. Infrared spectra were obtained using a Perkin- Elmer Spectrum BX instrument with a resolution of 4 cm^−1^ in the region of 400–4400 cm^−1^. A Micromeritics Gemini 2375 volumetric analyzer was used to measure the surface area using nitrogen physisorption isotherms. Thermogravimetric Analysis (TGA) was utilized on a Perkin-Elmer Pyris 1 TGA instrument with a temperature range of 25–1000 °C at a heating rate of 20 °C/min. In terms of X-ray photoelectron spectroscopy (XPS), the samples were analyzed by JPS-9030 JOEL. In a prior analysis, the sample was etched for 20 s by Ar gas in an Ultra High Vacuum Chamber (UHV) with pressure 10^−9^ torr. Malvern instruments (Zetasizer Nano ZS) was used to measure the particle size at different pH values. SpectraMax Plus 384 Microplate Reader was used to obtain UV spectra.

### 2.4. Drug Loading and Release

In the loading procedure: nanoparticles (1 mg) were dispersed in 1 mL of different concentration Dox solution and 1 mL of PBS. The suspension’s pH was adjusted to 3 by adding HCl (0.1 M) and stirred for 24 h at 25 °C in the dark condition. The pH change to 8 by adding NaOH (0.1 M) and stirred for 3 h. The solid was combined to determine the concentration of unloaded Dox [49]. The encapsulation efficiency (EE) and the loading capacity (LC) were estimated according to the following equations.

EE% = (Weight of loaded drug/Weight of drug in feed) × 100(1)

LC% = (Weight of loaded drug/Weight of Nanoparticles) × 100(2)

In the release procedure, 0.25 mg of Dox@Fe_3_O_4_@MSN-PDMAEMA or Dox @Fe_3_O_4_@MSN-PDMAEMA-FA was suspended at 37 °C in a buffer solution (1 mL) at a different pH under constant shaking (220 rpm). At a specific time, the nanoparticles were separated by centrifugation, the supernatant (1 mL) was removed, and the amount of released drug was determined by a UV-Vis spectrophotometer. The cumulative weight percent of Dox was calculated using the following equation.

(3) $$\mathrm{Cumulative}\;\mathrm{weight}\;(\%)\;=\;\frac{\mathrm{weight}\;\mathrm{of}\;\mathrm{drug}\;\mathrm{at}\;\mathrm{specific}\;\mathrm{time}\;\mathrm{points}\;}{\mathrm{weight}\;\mathrm{of}\;\mathrm{drug}\;\mathrm{in}\;\mathrm{nanoparticles}\;}\times 100$$

### 2.5. Cytotoxicity Assays

The cytotoxicity of PDEAEMA-modified hybrid magnetic nanoparticles is tested against cancer cell lines using a presto blue cell viability assay. Breast cancer cells (MCF-7) and resistant cancer cells (MCF-7 ADR) were harvested using 0.25% trypsin-EDTA. The cancer cells were subjected in 96-well plates at 3000 cells/well in DMEM media and cultured in 5% CO_2_ at 37 °C for 24 h. Various concentration of Free-DOX, Dox@Fe_3_O_4_@MSN-PDMAEMA, Dox @Fe_3_O_4_@MSN-PDMAEMA-FA, Fe_3_O_4_@MSN-PDMAEMA, and Fe_3_O_4_@MSN-PDMAEMA-FA were exposed to a cancer cell line in culture and incubated in 5% CO_2_ at 37 °C for 24 h.

## 3. Results and Discussion

Paramagnetic iron oxide nanoparticles (Fe_3_O_4_) were synthesized according to a co-precipitation protocol via mixing two types of iron salts. A SEM image (Figure S1) showed that the nanoparticles are of a spherical shape. Moreover, most of the Fe_3_O_4_ nanoparticles tend to form agglomerates before silica grows on their surface. The size distribution ranged between 10 and 20 nm, which agrees with Enache et al. [45].

**Figure 1:**
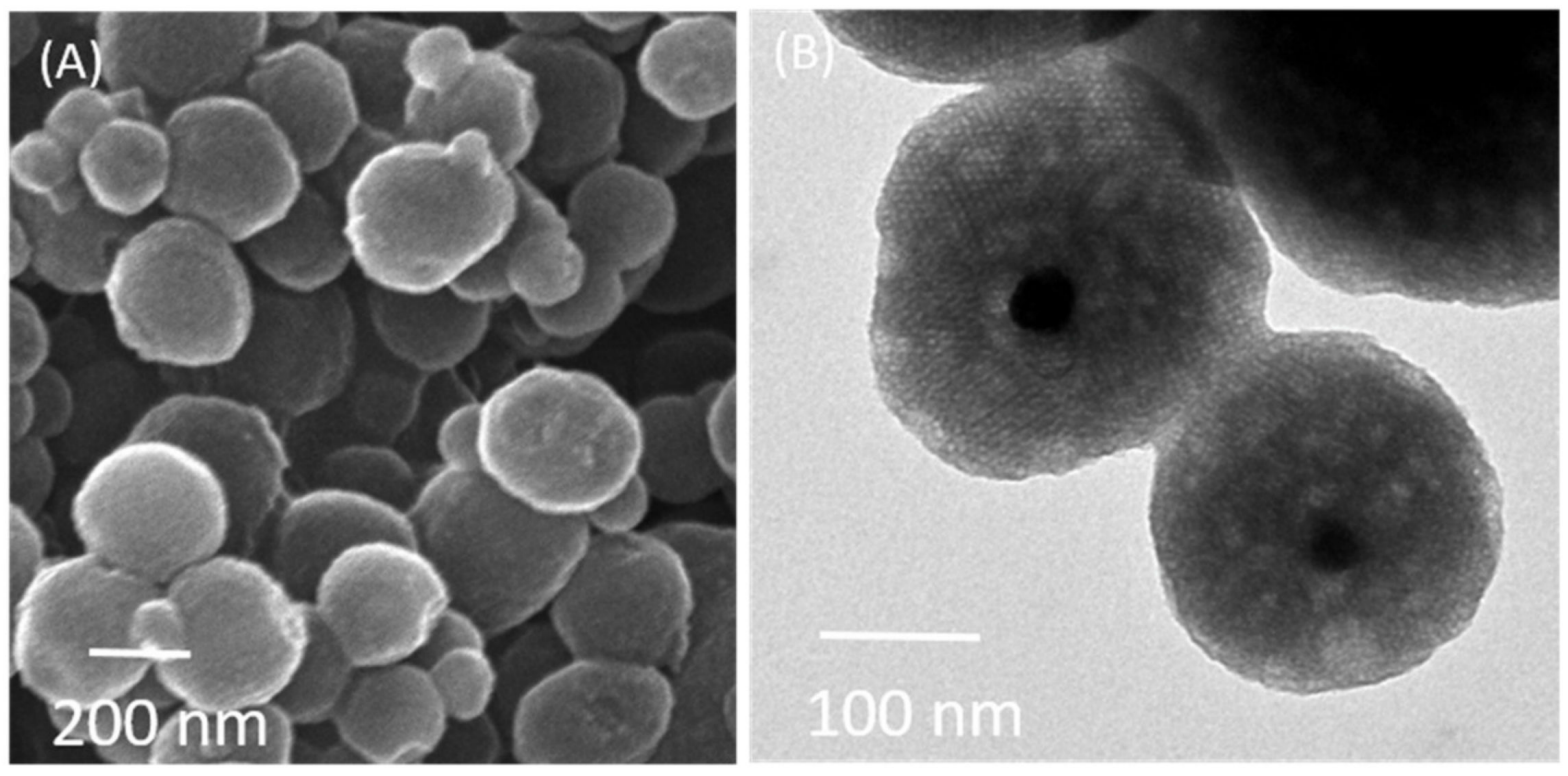
Microscopic images of magnetic mesoporous silica nanoparticles (Fe_3_O_4_@MSNs): (**A**) SEM image and (**B**) TEM images.

Fe_3_O_4_ nanoparticles were covered with silica in the presence of CTAB, forming a mesoporous shell (Figure 1). The SEM image showed that the shape of particles was spherical and semi-symmetrical with an average size ranging between 130 and 250 nm (Figure 1A). The TEM image was acquired for Fe_3_O_4_@MSNs as shown in Figure 1B. It can be seen that Fe_3_O_4_ nanoparticles were presented in the middle of Fe_3_O_4_@MSNs with a particle size of 20 nm, which agrees with the SEM image. The presence of surfactant could prevent Fe_3_O_4_ aggregation in the synthesis of the MSNs shell. The size of Fe_3_O_4_@MSNs was estimated to be ca. 200 nm.

**Figure 2:**
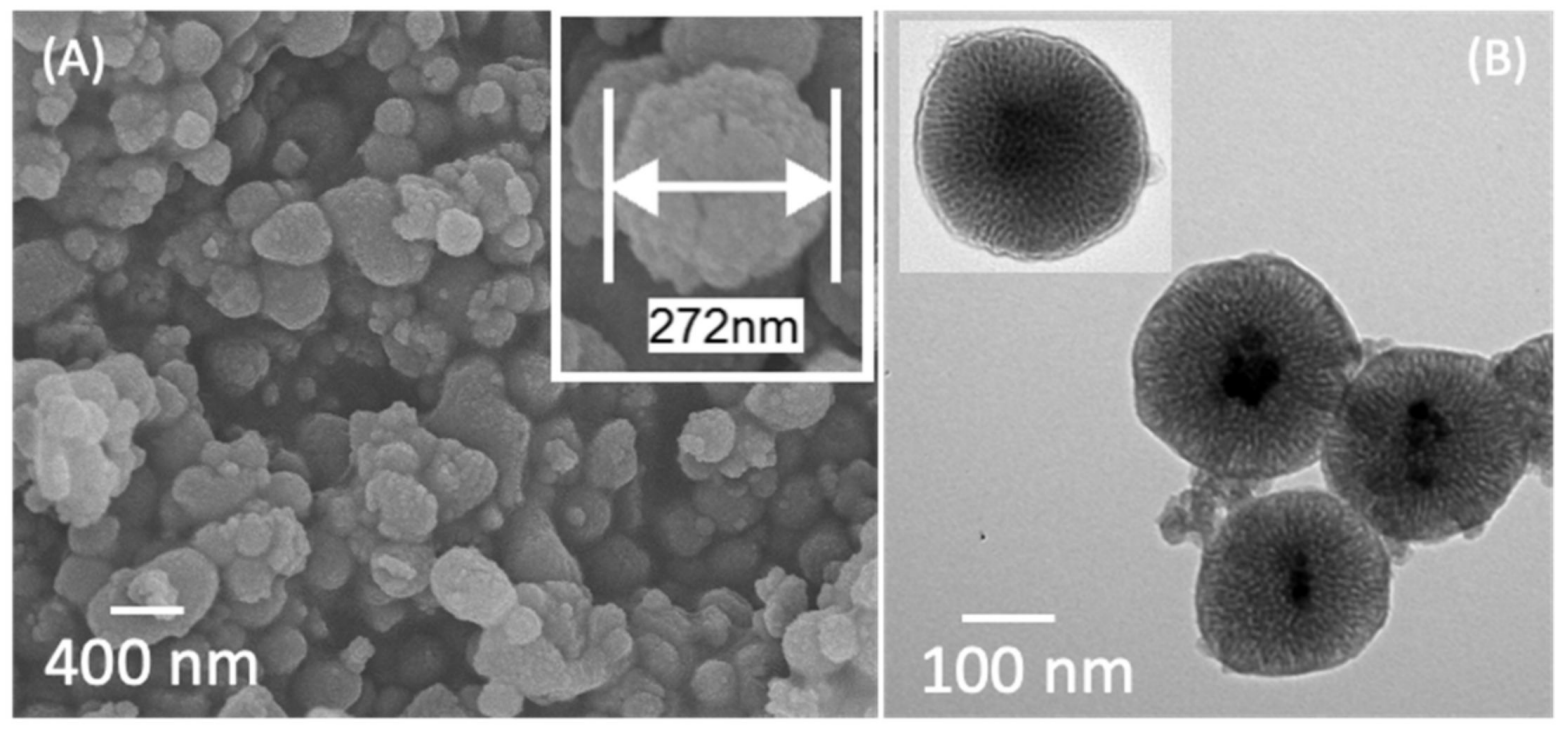
(**A**) SEM image and (**B**) TEM images of magnetic mesoporous silica nanoparticles coated with PDEAEMA.

Magnetic mesoporous silica nanoparticles were successfully coated with PDEAEMA brushes via SI-ATRP, as shown in SEM and TEM images (Figure 2). The average diameter of Fe_3_O_4_@MSN-PDEAEMA was ca. 250 nm with spherical morphology compared to 200-nm none-coated nanoparticles. PDEAEMA brushes’ thickness was estimated to be 50 nm, as illustrated in Figure 2A. The TEM image showed the size of Fe_3_O_4_@MSN-PDEAEMA was ca. 260 nm, which is in good agreement with the SEM image.

**Figure 3:**
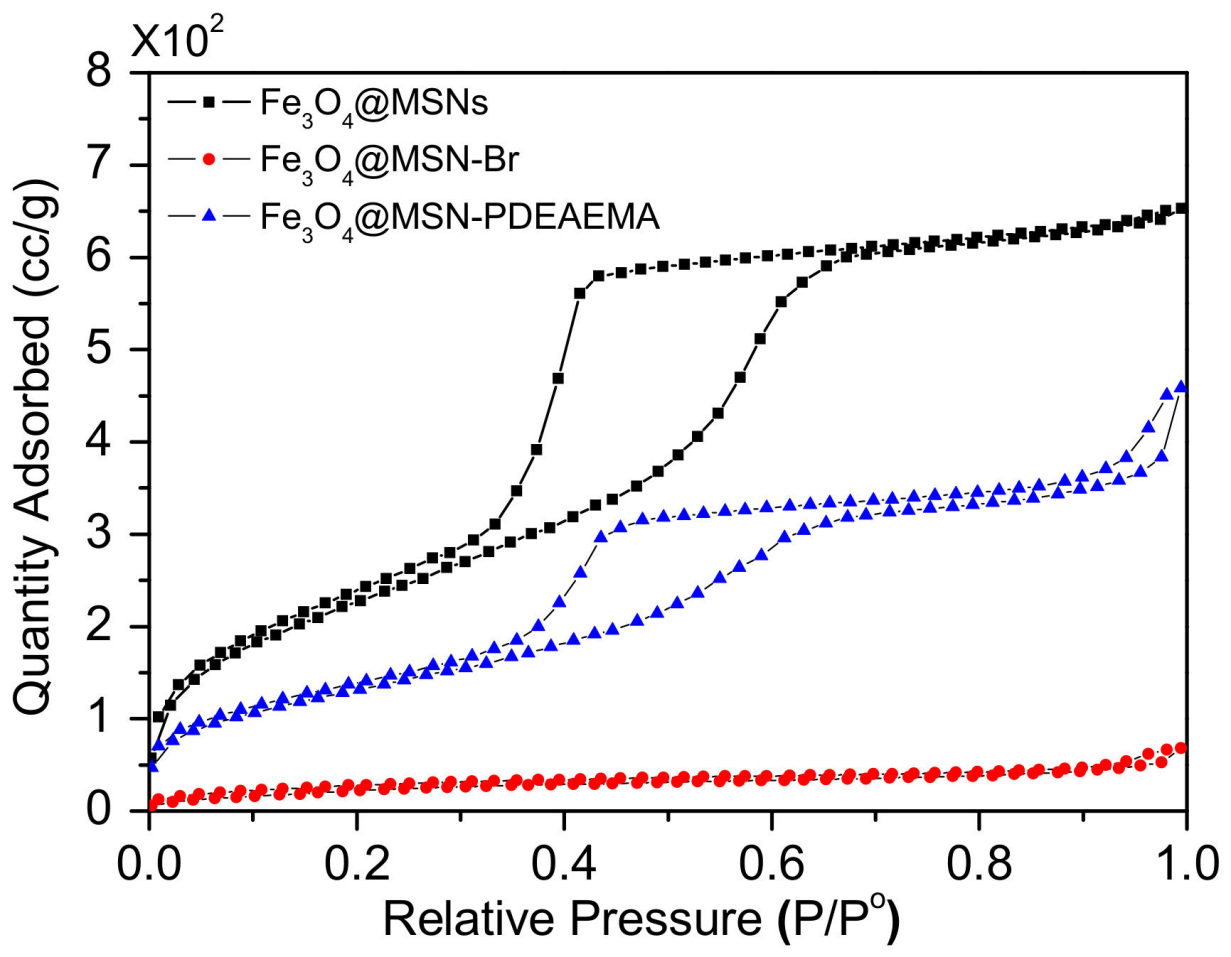
Brunauer–Emmett–Teller of Fe_3_O_4_@MSNs, Fe_3_O_4_@MSN-Br, and Fe_3_O_4_@MSN-PDEAEMA.

Brunauer–Emmett–Teller (BET) and Barrett–Joyner–Halenda (BJH) analysis were applied to determine the surface area and pore size of fabricated nanoparticles. Figure 3 reveals nitrogen adsorption-desorption isotherms for Fe_3_O_4_@MSNs, Fe_3_O_4_@MSN-Br, and Fe_3_O_4_@MSN-PDEAEMA. It can be clearly seen that Fe_3_O_4_@MSNs and Fe_3_O_4_@MSN-Br exhibit type IV physisorption isotherm, indicating mesopores are present in the former. The surface area and pore volume of the Fe_3_O_4_@MSNs were found to be 722 m^2^·g^−1^, 0.92 cm^3^·g^−1^, respectively. The specific surface area was 489 m^2^·g^−1^ for Fe_3_O_4_@MSN-Br, as illustrated in Table S1. Fe_3_O_4_@MSN-PDEAEMA exhibits an almost type II isotherm with surface area of 82 m^2^·g^−1^. It would be reasonable to assume that the presence of dense polymer layers on the Fe_3_O_4_@MSN surface could prevent penetration of the N_2_ gas due to the existence of ATRP initiating groups on the exterior surface of Fe_3_O_4_@MSN [50,51].

FTIR spectra of Fe_3_O_4_@MSNs (with CTAB), Fe_3_O_4_@MSNs, Fe_3_O_4_@MSN-Br, and Fe_3_O_4_@MSN-PDEAEMA are presented in Figure S2. For all samples, the absorption peaks at 1084 and 1230 cm^−1^ were referred to as Si-O-Si bonds. The absorption peaks at 3450 and 960 cm^−1^ were assigned to the stretching absorption vibration of Si-OH. Fe_3_O_4_@MSNs (with CTAB), C–H stretching bands were presented at 2930 cm^−1^ and 2860 cm^−1^. These peaks disappeared after CTAB extraction. After a polymerization process, a new peak appears at 1720 cm^−1^ corresponding to C=O groups compared to Fe_3_O_4_@MSN-Br.

**Figure 4:**
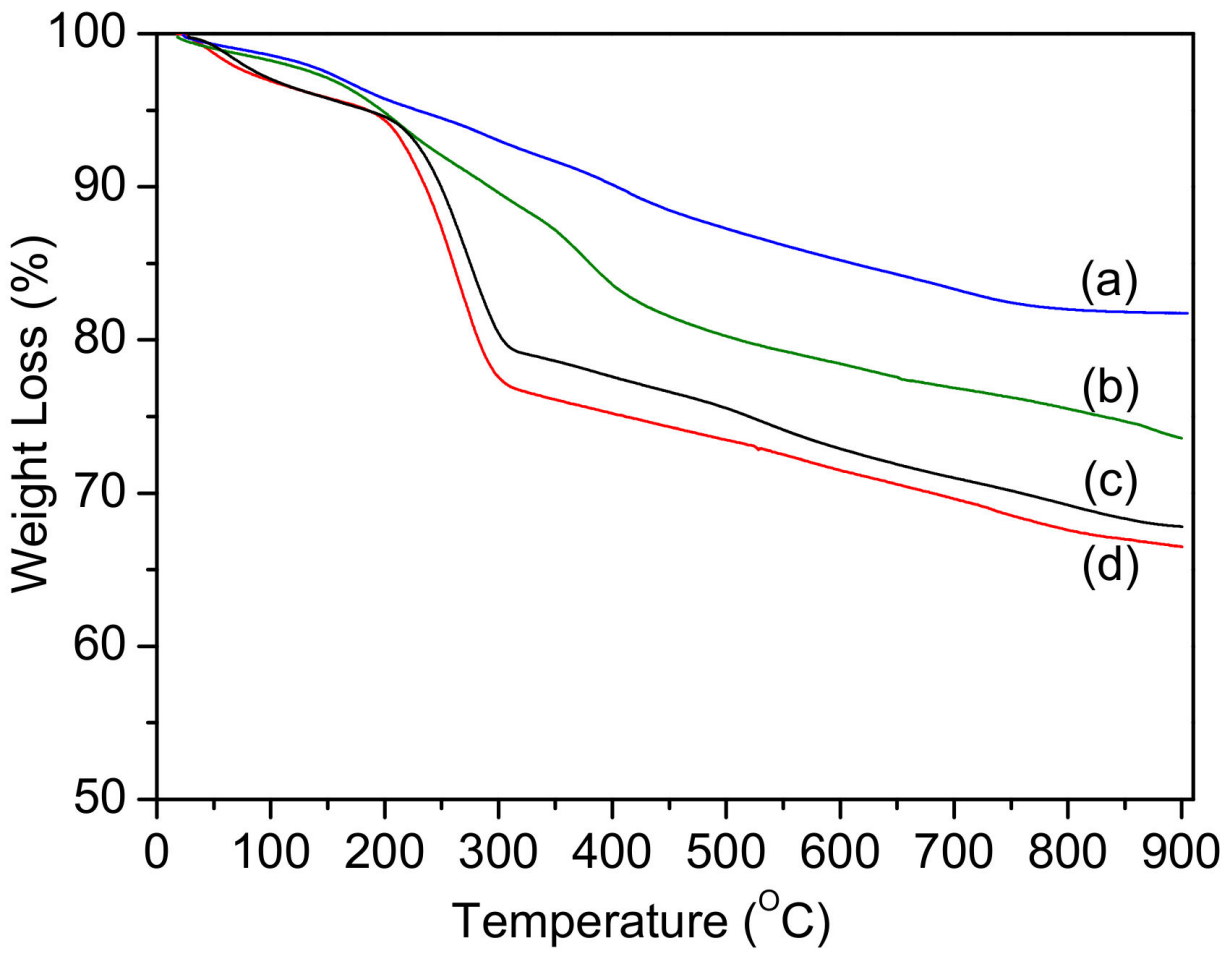
TGA curves of Fe_3_O_4_@MSN-NH_2_ (with CTAB) (c), Fe_3_O_4_@MSN-Br (with CTAB) (d), Fe_3_O_4_@MSN-Br (a), and Fe_3_O_4_@MSN-PDEAEMA (b).

The successful decoration of magnetic mesoporous silica was evaluated by thermogravimetic analysis (TGA) when heating in an N_2_ atmosphere to 1000 °C. It was noticed that there was an increase in the weight loss after each synthetic step, which confirms the surface modification of Fe_3_O_4_@MSNs (Figure 4) [37,52,53]. The weight loss of Fe_3_O_4_@MSN-NH_2_ with the surfactant has three mass loss steps. The first step started from the ambient to ca. 215 °C, which ascribed to the water desorption and dehydration from nanoparticles. The second loss step was from ca. 230 to 320 °C, which was assigned to the degradation of the surfactant and organic groups in a silane monolayer. The third step started from ca. 350 °C, which attributed to the nanoparticles’ dehydroxylation. The total weight loss of Fe_3_O_4_@MSN-NH_2_ with CTAB was estimated to be ca. 28%. Similar behavior was observed when the amino functionalized Fe_3_O_4_@MSN-NH_2_ with CTAB reacted with BIBB. The weight loss of initiated nanoparticles was ca. 27% after removing CTAB. The surfactant weight in the nanoparticles was estimated to be ca. 13%. After the growth of PDEAEMA, the weight loss was three mass loss steps. The first step started from the ambient to ca. 250 °C, corresponding to the water desorption and dehydration. The second loss mass step ranged from ca. 300 to 450 °C, which was assigned to the decomposition of PDEAEMA chains. The last weight loss step was around 480 °C, which attributed to the nanoparticles’ dehydroxylation. The polymer weight attached to Fe@MSNs’ surface was estimated to be ca. 9%.

**Figure 5:**
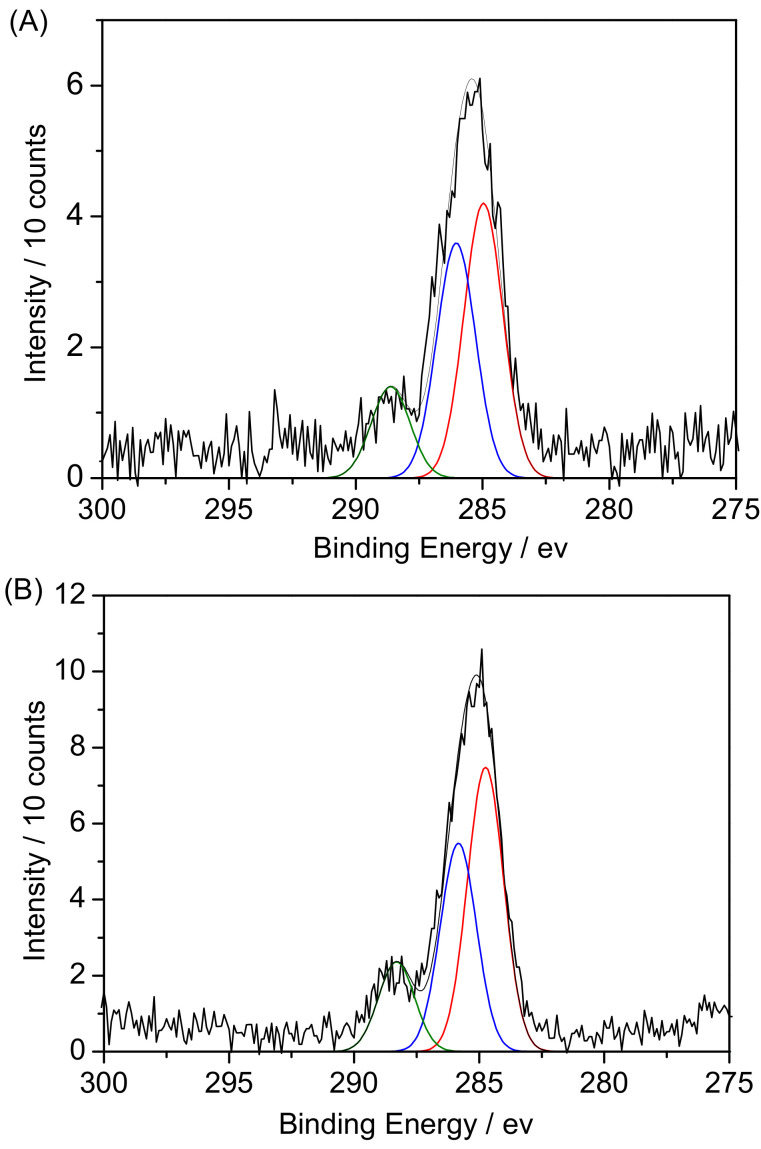

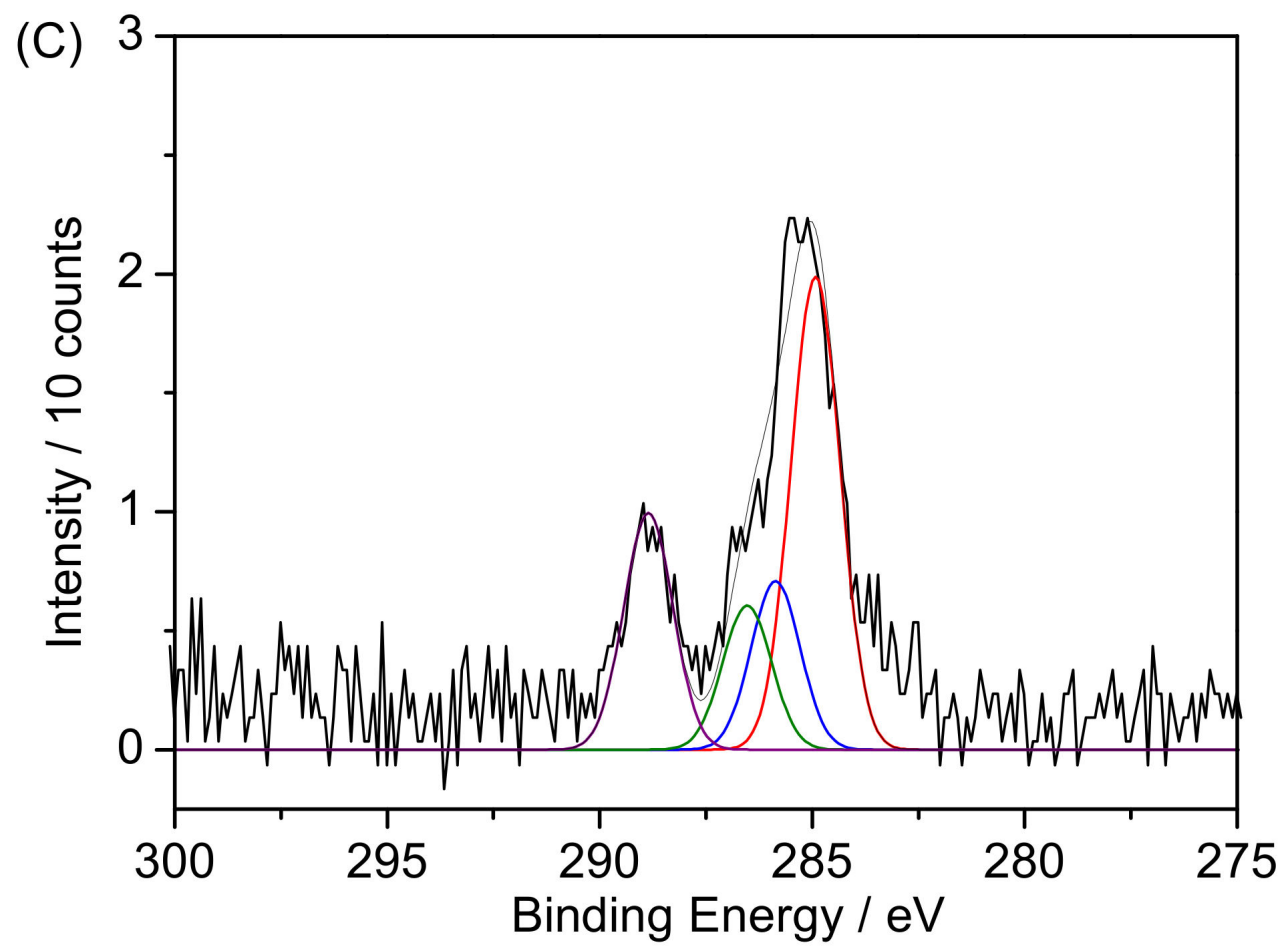
XPS spectra of C1s peaks for: (**A**) Fe_3_O_4_@MSN-PDEAEMA, (**B**) Fe_3_O_4_@MSN-PDEAEMA-NH_2_, and (**C**) Fe_3_O_4_@MSN-PDEAEMA-FA.

X-ray photoelectron spectroscopy was performed to study the surface modification of Fe_3_O_4_@MSN-PDEAEMA (Figure 5). The C1s spectrum of the PDEAEMA brush grafted on Fe_3_O_4_@MSNs was fitted with three peaks at 284.8 eV, 286.1 eV, and 288.6 eV with a ratio of 4.5:3.8:1, corresponding to the C–H, C– (N, O), and O=C–O, respectively, in good agreement with a theoretical ratio of 5:4:1 (Figure 5A) [52,53]. After aminating the end group of the polymer chains, there is no difference in C1s spectrum of PDEAEMA, as shown in Figure 5B. After surface derivatization with folic acid, a peak was fitted at ~286.5 eV, corresponding to the C–O bonds’ present organic layer. Moreover, the peak intensity at 288.5 eV increased, which confirmed the successful attachment of folic acid (Figure 5C).

**Figure 6:**
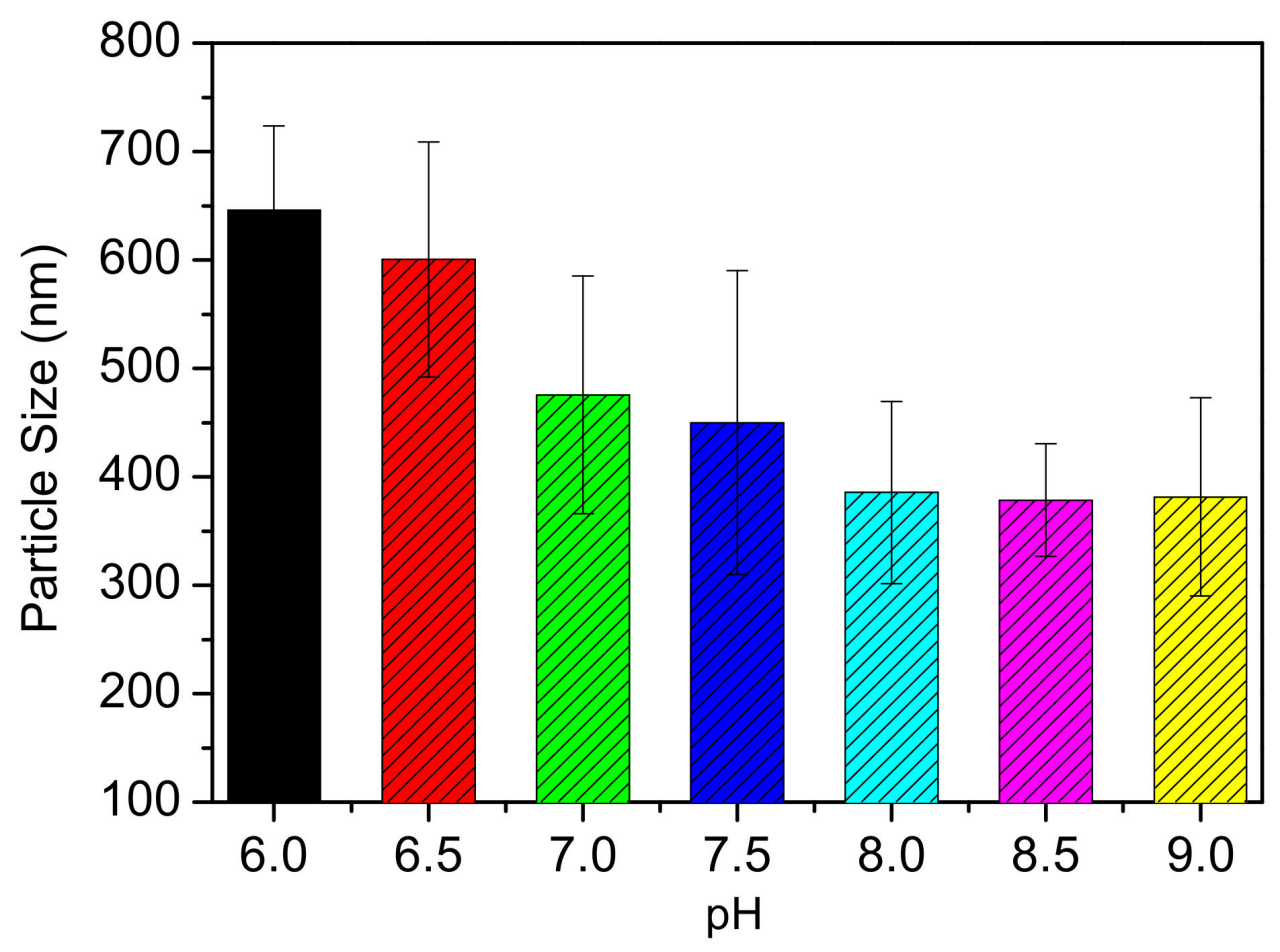
Illustration the change in size of Fe_3_O_4_@MSN-PDEAEMA as function of pH value at 20 °C.

Hydrodynamic sizes of Fe_3_O_4_@MSN-PDEAEMA was measured in PBS at different pH values by DLS, as shown in Figure 6. The size of Fe_3_O_4_@MSN-PDEAEMA change as the pH values change due to the protonation or deprotonation of amino groups at PDEAEMA chains. Fe_3_O_4_@MSN-PDEAEMA hydrodynamic size increased from ca. 550 nm (at pH 7) to 750 nm (at pH 4) due to the protonation process of amino groups causing a hydrophilic characteristic. In an alkaline solution, the amino groups in PDEAEMA segments were deprotonated and became a hydrophobic polymer, leading to a decrease in the size of particles from ca. 550 nm to ca. 350 nm at pH 8.

Fe_3_O_4_@MSN-PDEAEMA and Fe_3_O_4_@MSN-PDEAEMA-FA loading capacities were measured by UV–Vis spectroscopy at 480 nm. At pH = 3, PDEAEMA chains are protonated and expanded, leading to open the pores and allow Dox to pass into the pores. At pH = 8, PDEAEMA chains collapsed and were hosted into pores. As observed, the amounts of drug storage increased as Dox concentrations increased. At the same concentration, there was no significant difference between Fe_3_O_4_@MSN-PDEAEMA and Fe_3_O_4_@MSN-PDEAEMA-FA in the loading capacity (see Table S2 and Figure S3).

**Figure 7:**
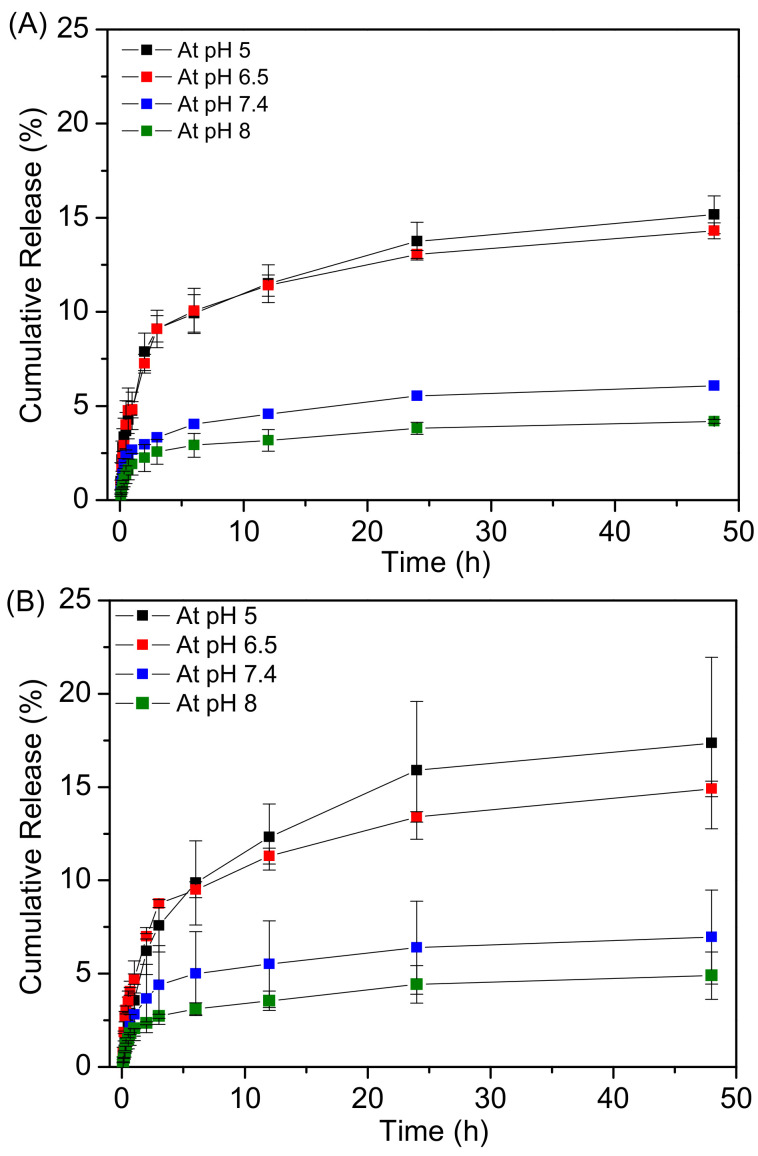
Drug release pattern of DOX loaded in: (**A**) Fe_3_O_4_@MSN-PDEAEMA and (**B**) Fe_3_O_4_@MSN-PDEAEMA-FA in different pH values at 37 °C.

The drug release from Fe_3_O_4_@MSN-PDEAEMA and Fe_3_O_4_@MSN-PDEAEMA-FA were investigated at different pH buffer solutions. The results show that Dox’s cumulative release from both nano-systems was higher in acidic solution than basic media (Figure 7). At pH > 7, the amount of drug release was less than 6% within 48 h, due to the collapse process of the PDEAEMA segments. After 48 h, an insignificant difference in cumulative Dox release was observed for both nano-systems at a pH of 5 and 6.5, which was ca. 16% and ca. 13%, respectively. Protons in the solution can diffuse through PDEAEMA chains easily and protonate the drug’s amino group, accelerating the drug release.

**Figure 8:**
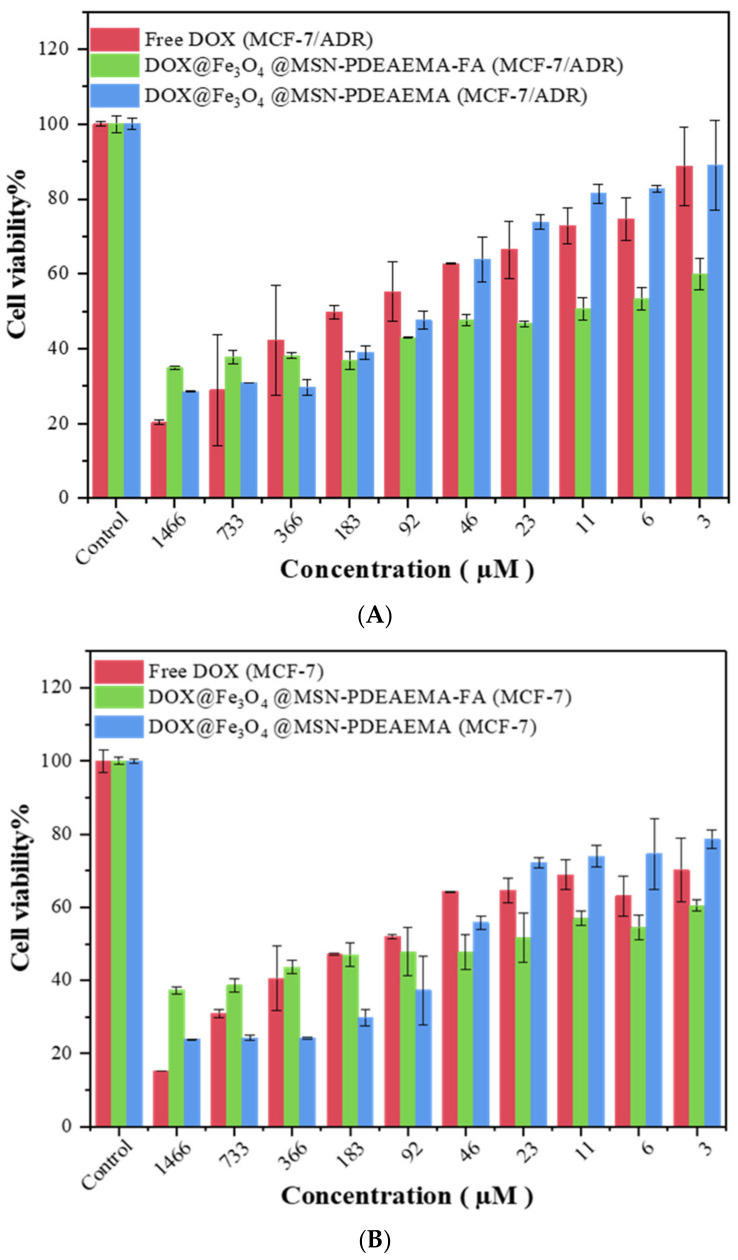
Cytotoxicity assay of the hybrid magnetic nanostructured. (**A**) Exposure of Free-DOX, Dox@Fe_3_O_4_@MSN-PDMAEMA, and Dox @Fe_3_O_4_@MSN-PDMAEMA-FA to MCF-7-ADR cells for 24 h. (**B**) Exposure of Free-DOX, Dox@Fe_3_O_4_@MSN-PDMAEMA, and Dox @Fe_3_O_4_@MSN-PDMAEMA-FA to MCF-7 cells for 24 h.

The cytotoxicity effect of Exposure of Free-DOX, Fe_3_O_4_@MSN-PDMAEMA, Fe_3_O_4_@MSN-PDMAEMA-FA, Dox@Fe_3_O_4_@MSN-PDMAEMA, and Dox @Fe_3_O_4_@MSN-PDMAEMA-FA was assessed using MCF-7-ADR cells MCF-7 cells (Figure 8). No cytotoxic effect was observed against MCF-7-ADR cells and MCF-7 cells when unloaded nano-systems were exposed to the cells, even at a high concentration, as shown in Figure S4. Folic acid modified Fe_3_O_4_@MSN-PDMAEMA mitigated the cytotoxic effects. Considerable cell damage or cell death when Free-DOX, Dox@Fe_3_O_4_@MSN-PDMAEMA, or Dox @Fe_3_O_4_@MSN-PDMAEMA-FA was exposed with MCF-7-ADR cells MCF-7 cells for 24 h (Figure 8A,B). It is clear that a significant decrease in cell viability was found at all concentrations of Free-DOX, Dox@Fe_3_O_4_@MSN-PDMAEMA, or Dox @Fe_3_O_4_@MSN-PDMAEMA-FA in the cell lines. Cell viability systematically decreased with increasing concentration of Free-DOX, Dox@Fe_3_O_4_@MSN-PDMAEMA, or Dox@Fe_3_O_4_@MSN-PDMAEMA-FA. However, a low level (>60%) of cell viability can be seen only in a very low concentration of Dox@Fe_3_O_4_@MSN-PDMAEMA-FA, compared to Free-DOX, Dox@Fe_3_O_4_@MSN-PDMAEMA. At a high concentration, Dox@Fe_3_O_4_@MSN-PDMAEMA show the higher cytotoxic effect than Dox@Fe_3_O_4_@MSN-PDMAEMA-FA in MCF-7-ADR and MCF-7 cell lines.

## 4. Conclusions

In summary, we reported folic acid decorated the surface of PDEAEMA-grafted on magnetic mesoporous nanoparticles (Fe_3_O_4_@MSN-PDEAEMA) and modified with folic acid (Fe_3_O_4_@MSN-PDEAEMA-FA) for biomedical application. First, Fe_3_O_4_ cores were coated with mesoporous silica (average size of 180 nm), followed by surface functionalized with an ATRP initiator. PDEAEMA brushes were grafted with a dry thickness of 50 nm, via the AGET ATRP technique. The end-groups of PDEAEMA brushes were converted to amines s using sodium azide and triphenylphosphine. Folic acid was activated using EDC and NHS, and reacted with Fe_3_O_4_@MSN-PDEAEMA-NH_2_. The characteristic of the nanoparticles was identified using SEM, TEM, FT-IR, TGA, and XPS. The nanoparticle’s size increased from ca. 350 nm in basic media, to ca. 750 nm in acidic media due to the protonation of tertiary amine in the PDEAEMA, as estimated by DLS. The anti-cancer drug (DOX) was loaded within Fe_3_O_4_@MSN-PDEAEMA and Fe_3_O_4_@MSN-PDEAEMA-FA with ca. 66% loading capacity. The drug release pattern illustrates that Dox was released in a relative control manner from both nano-systems.

## Data Availability

The data presented in this study is openly available.

## Supplementary Materials

The following are available online at https://www.mdpi.com/2073-4360/13/1/59/s1. Further details of SEM image of Fe_3_O_4_, table of physiochemical data for decorated nanoparticles, FTIR spectra, Dox loading capacity of fabricated nanoparticles, and loading capacities of fabricated nanoparticles.
