# Supplementary material for: Folic Acid-Terminated Poly(2-Diethyl Amino Ethyl Methacrylate) Brush-Gated Magnetic Mesoporous Nanoparticles as a Smart Drug Delivery System
Source: Polymers (Basel). 2020 Dec 25;13(1):59. doi: 10.3390/polym13010059 (PMC7795197; doi:10.3390/polym13010059)
Supplement: Supplementary file 1 [file polymers-13-00059-s001.pdf]

Folic acid- terminated poly (2- diethyl amino ethyl methacrylate) brush - gated magnetic mesoporous nanoparticles as smart drug delivery system

Abeer M Beagan,[1‡*] Ahlam A Alghamdi,[1] Shatha S Lahmadi,[1] Majed A. Halwani,[2] Mohammed S Almeataq,[3] Abdulaziz Alhazaa,[4] Khalid Alotaibi and Abdullah M. Alswieleh [1‡*]

[1] Chemistry Department, College of Science, King Saud University, Riyadh, Kingdom of Saudi Arabia.

[2] Nanomedicine Department, King Abdullah International Medical Research Center, King Saud bin Abdulaziz University for Health Sciences, Riyadh, Kingdom of Saudi Arabia.

[3] King Abdulaziz City for Science and Technology, Riyadh, Saudi Arabia.

[4] Research chair for Tribology, Surface and Interface Science (TSIS), Physics and Astronomy Department, College of Science, King Saud University, Riyadh, Kingdom of Saudi Arabia

* Corresponding Author: A. Alswieleh, Email: aswieleh@ksu.edu.sa

A. Beagan, Email: abeagan@ksu.edu.sa

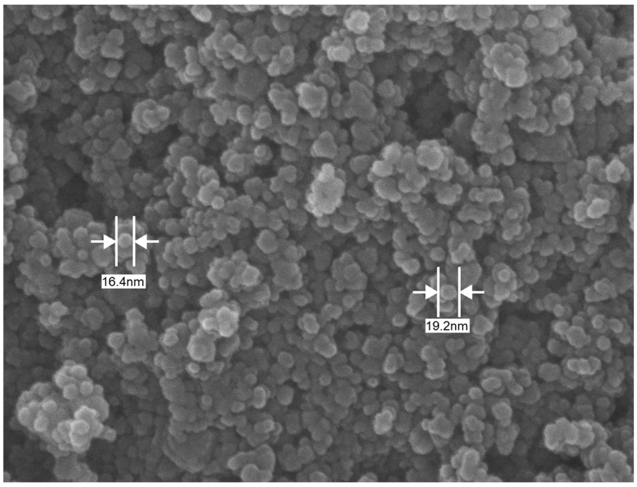

Figure S1: SEM image of paramagnetic iron oxide nanoparticles (Fe_3O_4).

Table S1: Physiochemical data obtained for decorated nanoparticles.

Sample	BET surface area ($m^2.g^{-1}$)	Pore volume ($cm^3.g^{-1}$)
Fe_3O_4@MSNs	722	0.92
Fe_3O_4@MSN-Br	489	0.58
Fe_3O_4@MSN-PDEAEMA	82	0.07

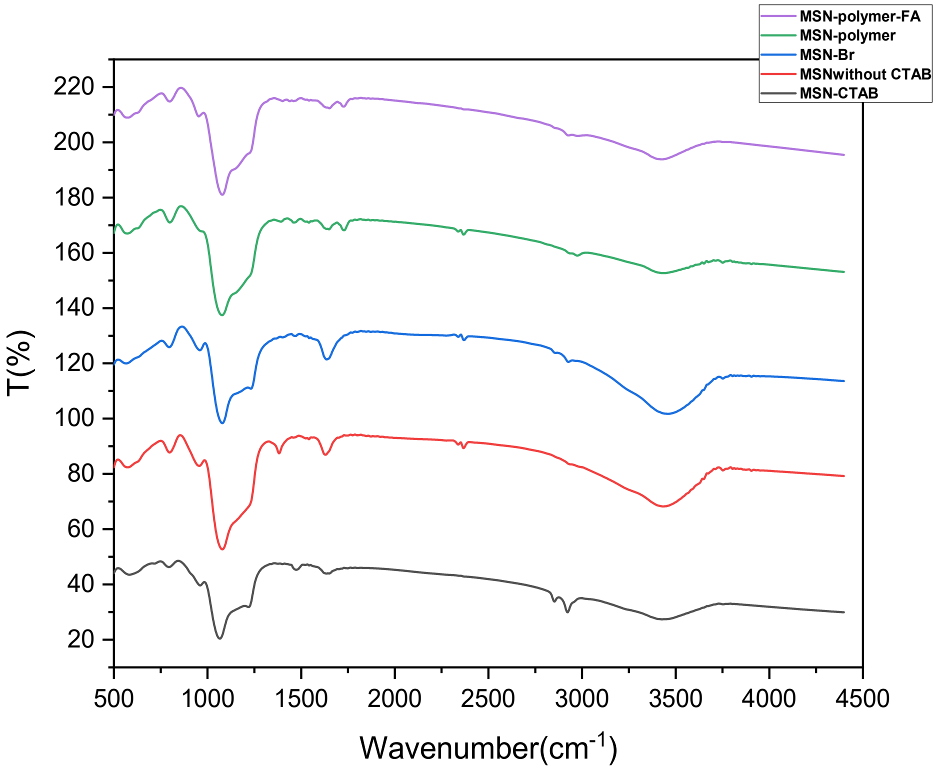

Figure S2: FTIR of spectra of Fe_3O_4@MSNs (with CTAB), Fe_3O_4@MSNs, Fe_3O_4@MSN-Br and Fe_3O_4@MSN-PDEAEMA.

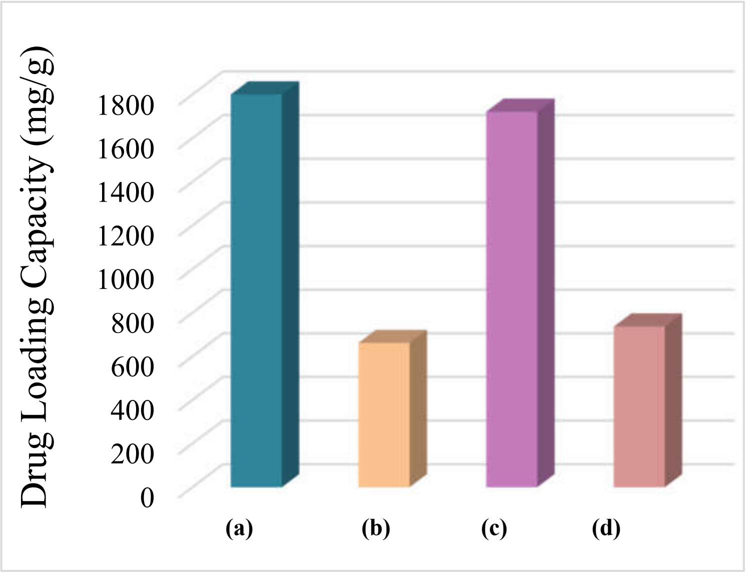

Figure S3: Dox loading Capacity for: a) Fe_3O_4@MSN-PDEAEMA (1000 ppm), b) Fe_3O_4@MSN-PDEAEMA (500 ppm), c) Fe_3O_4@MSN-PDEAEMA-FA (1000 ppm), d) Fe_3O_4@MSN-PDEAEMA-FA (500 ppm).

Table S2: Dox loading Capacity and Entrapment Efficiency for: a) Fe_3O_4@MSN-PDEAEMA (1000 ppm), b) Fe_3O_4@MSN-PDEAEMA (500 ppm), c) Fe_3O_4@MSN-PDEAEMA-FA (1000 ppm), d) Fe_3O_4@MSN-PDEAEMA-FA (500 ppm).

Sample	*Loading Capacity %*	*Entrapment Efficiency %*
Fe_3O_4@MSN-PDEAEMA (1000 ppm)	64. 3%	90.03%
b) Fe_3O_4@MSN-PDEAEMA (500 ppm)	39.83%	66.2%
Fe_3O_4@MSN-PDEAEMA-FA (1000 ppm)	63.25%	86.05%
Fe_3O_4@MSN-PDEAEMA-FA (500 ppm)	42.43%	73.72%

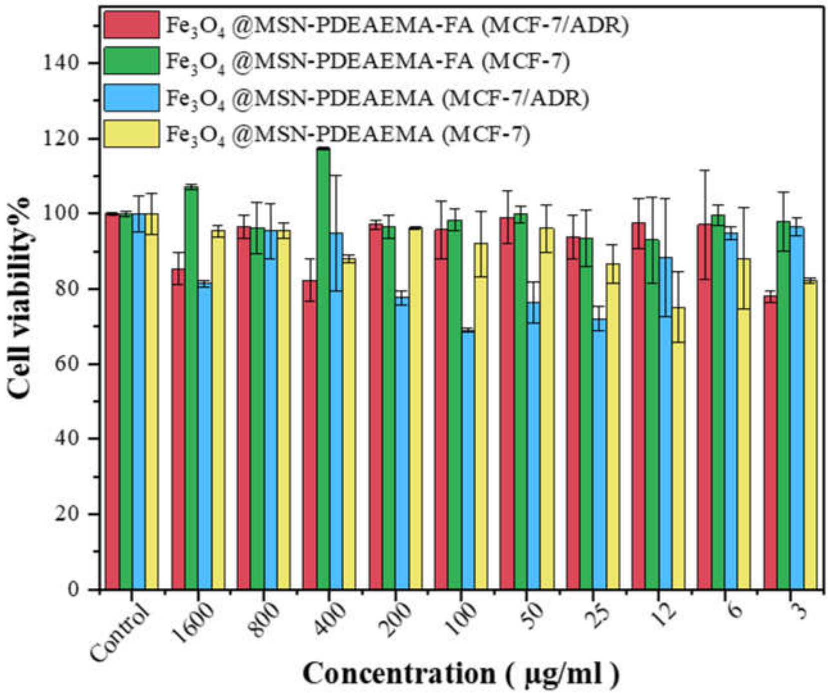

Figure S4. Cytotoxicity assay of the hybrid magnetic nanostructured. (A) Exposure of Fe_3O_4@MSN-PDMAEMA and Fe_3O_4@MSN-PDMAEMA-FA to MCF-7-ADR and MCF-7 cell for 24 h.
